# Social Determinants of Health in Physiatry: Challenges and Opportunities for Clinical Decision Making and Improving Treatment Precision

**DOI:** 10.3389/fpubh.2021.738253

**Published:** 2021-11-11

**Authors:** Rosalynn R. Z. Conic, Carolyn Geis, Heather K. Vincent

**Affiliations:** ^1^Department of Family Medicine and Public Health, University of California, San Diego, San Diego, CA, United States; ^2^Department of Physical Medicine and Rehabilitation, University of Florida, Gainesville, FL, United States

**Keywords:** big data, physical function, outcomes, physiatry, physical medicine and rehabilitation, social determinants of health

## Abstract

Physiatry is a medical specialty focused on improving functional outcomes in patients with a variety of medical conditions that affect the brain, spinal cord, peripheral nerves, muscles, bones, joints, ligaments, and tendons. Social determinants of health (SDH) play a key role in determining therapeutic process and patient functional outcomes. Big data and precision medicine have been used in other fields and to some extent in physiatry to predict patient outcomes, however many challenges remain. The interplay between SDH and physiatry outcomes is highly variable depending on different phases of care, and more favorable patient profiles in acute care may be less favorable in the outpatient setting. Furthermore, SDH influence which treatments or interventional procedures are accessible to the patient and thus determine outcomes. This opinion paper describes utility of existing datasets in combination with novel data such as movement, gait patterning and patient perceived outcomes could be analyzed with artificial intelligence methods to determine the best treatment plan for individual patients in order to achieve maximal functional capacity.

## Introduction

**Figure 1:**
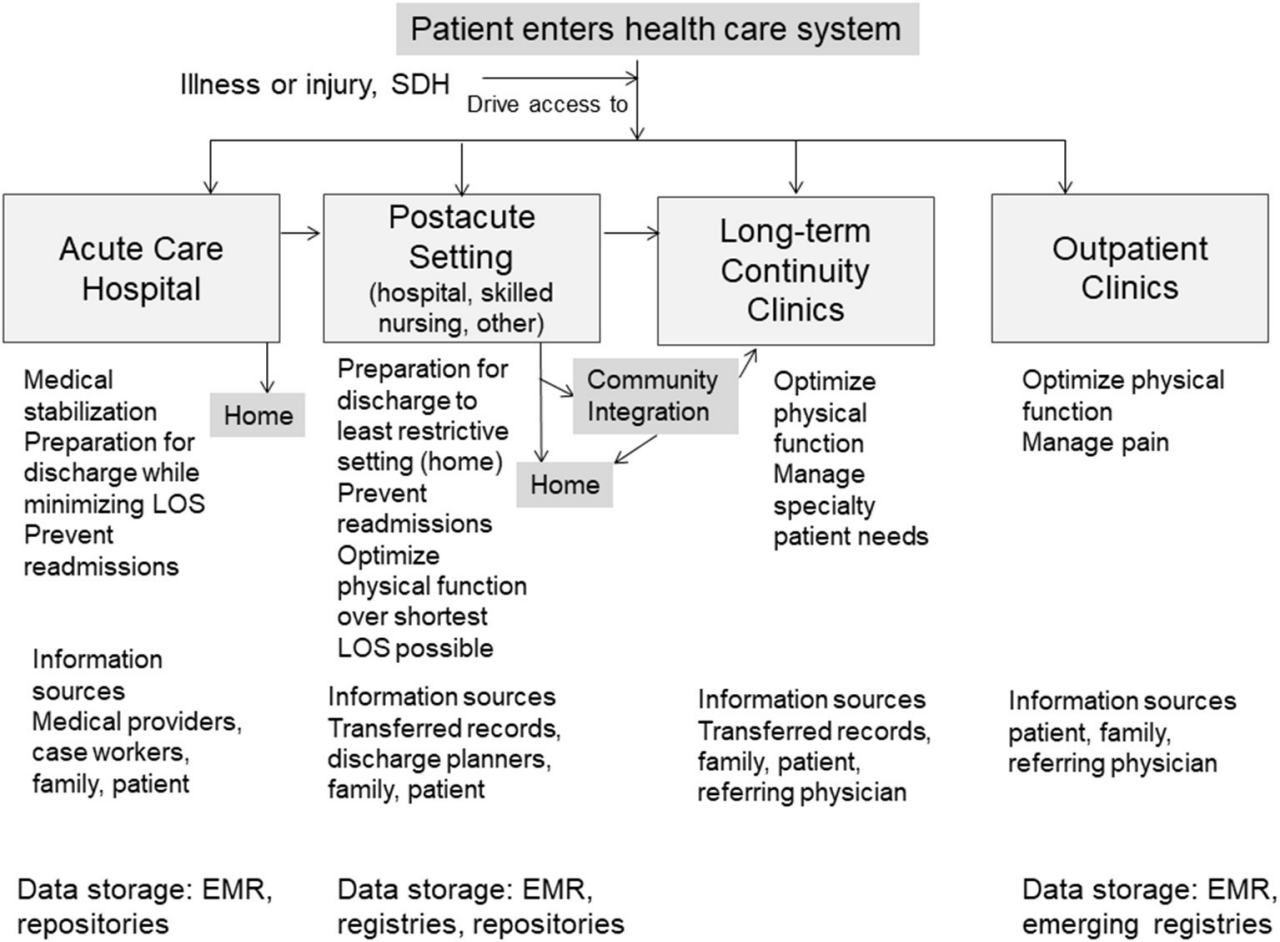
Overview of possible patient pathways through the spectrum of physiatric care and variation of medical data sources and SDH captured and stored across rehabilitation settings.

Physical medicine and rehabilitation, or physiatry, is a specialty that treats medical conditions affecting the brain, spinal cord, peripheral nerves, joints, muscle, bone, tendons and ligaments. The main treatment goal of physiatry is to maximize function and independent living. Physiatry care spans the entire continuum of health care from consultation in the acute care hospital to post-acute inpatient rehabilitation, home health, outpatient, and community re-integration. Patients move through these levels of care as they gain functional independence or have a need for ongoing care (Figure 1). Patients enter the healthcare system at different “starting points” in the care spectrum. At each phase of care and transition, physiatrists coordinate patient care and make critical decisions regarding rehabilitation needs based on medical status and functional progress. This decision-making is made more complex by the wide diversity of patient types, socioeconomic backgrounds, medical conditions, injury complexity, and patient-family perception of needs.

Social determinants of health (SDH) are various social and economic factors, including education, healthcare access, and community support, which can impact health status and health outcomes (1). SDH guide resource allocation, discharge planning, access to outpatient rehabilitation services and other therapeutic interventions and progress assessment. However, despite their potential impact, SDH data are notoriously poorly collected and coded in the electronic health record (EHR) (2) which makes assessing their impact *post-hoc* challenging. Furthermore, specific SDH can contribute to disparities in outcomes among patient subgroups (3, 4). For example, SDH, including educational attainment, housing and living environment, and social support, influence rehabilitation outcomes with various post-acute conditions such as stroke (5–14), spinal cord injury (SCI) (15, 16), traumatic brain injury (TBI) (17–21), amputation (22–24), and chronic conditions such as osteoarthritis (25), chronic pain (26), and cardiopulmonary disease (27).

Due to wide availability of therapies and interventions, it is challenging for physiatrists to determine which patient subgroups will achieve the best outcomes. This challenge may be met through exploration of big data and artificial intelligence techniques. Presently, machine learning and artificial intelligence are not commonly used in this field to predict outcomes, but should be. We propose a critical reappraisal of data collection methods and development of a “biopsychosocial model” (28) that includes SDH and physical functional measures. In this opinion and perspective paper, we present: (1) SDH driving functional outcomes; (2) available big datasets relevant to physiatry and possible artificial intelligence application; and (3) new measurement and analysis methods that could improve care pathway mapping and functional outcomes in physiatry. The search terms “social determinants of health,” “big data,” “electronic health record,” “physiatry,” “rehabilitation,” “physical medicine and rehabilitation” were used to identify relevant articles discussed herein. All relevant articles were reviewed and representative articles that included the main patient populations treated in physiatry are presented next.

## Social Determinants of Health on Physiatry Care Pathways

SDH are vital to collaborative short and long-term goal setting with the patient and family, with establishing home safety parameters, setting expectations for rate and type of functional gains, and reintegration into social-vocational roles. In the outpatient setting, SDH affects symptom progression, mental health, social functioning and access to the amount or type of services obtained for a given diagnosis (29). Commonly measured SDH each care setting are summarized in Supplementary Table 1.

### Acute Care Setting

In acute care, SDH are reviewed that could impact referral decisions and admissions into post-acute care. The decision to refer is described as “subjective” (30), yet referral of patients to the appropriate level of care ensures equitable access (31). Limiting or delaying access to services after severe injuries such as stroke or TBI can worsen functional disability and related outcomes^27^ and contributes to health disparities. For many conditions, early intensive rehabilitation can optimize functionality and re-engage patients back into life. SDH that affect referral to post-acute services include gender, race (29, 32), age, payor source (32), place of living (community alone, community with others, nursing home) (30), social support or living status, and geographic region (23). For medically-complex conditions, such as dysvascular amputation, inpatient rehabilitation referrals occur more often when the patient is married, has Medicaid and lives in a city; older, unmarried patients with history of nursing home residence are more often referred to skilled nursing facilities (SNF) (23). Patients with knee or hip arthroplasty may enter the rehabilitation pathway in post-acute care or outpatient settings depending on SDH, including age, gender and availability of caregiver at home. Younger patients and those with more family support are commonly referred to less intensive care settings (33). Among patients with hip fracture or joint replacement, SNF placement was more common in those with no insurance, Medicaid, and those who were Hispanic or black. SNFs are associated with less rigorous rehabilitation compared to an inpatient rehabilitation hospital (34, 35). Thus, a key transition at which functional outcomes is impacted is discharge to the next setting.

### Post-acute Care Setting

The post-acute care setting shapes functional and clinical outcomes by rehabilitation prescription (type and volume of therapies). Inpatient rehabilitation hospitals are required to provide physician management at least 3 days per week, 24 h nursing care and at least 3 h of intensive rehabilitation therapy five times a week. Differences exist in the delivery of occupational, physical and speech-language therapy among post-acute settings for treatment of the same diagnosis (36). Gains in mobility and self-care are frequently better after inpatient rehabilitation compared to SNF (36, 37). Unfavorable outcomes in post-acute settings include long rehabilitation hospital stays, slow trajectory to achieve functional milestones (mobility, various activities), small functional gains, discharge to long-term care and acute care readmission. In general, worse outcomes occur with advanced age (15, 38–40), non-white race (19, 41), insurance type (42), less family support or living alone (23, 32, 40). Older patients are less able to engage in intensive rehabilitation therapies for SCI or hip fracture (3, 15). Some SDH, such as gender, have differential effects on rehabilitation outcomes. Specifically, female gender is associated with higher odds of discharge to home (43) and better supervision-level only status for more functional activities than men after stroke by discharge (10, 43, 44), but females demonstrate lower efficiency of functional improvement during rehabilitation than males after knee arthroplasty (45).

Readmission to acute care is differentially affected by SDH in different settings. For patients with knee arthroplasty receiving care in an inpatient rehabilitation hospital, advanced age and non-white race increased the odds for 90-day readmission (35). However, age, gender, race, marital status and living arrangement did not predict hospital readmissions for patients in a SNF, but medical conditions such as congestive heart failure did (46). Other evidence shows that patients with SCI are more likely to be readmitted multiple times if unemployed, female, have Medicaid (16, 47) or if rehabilitation was provided in a SNF (48). SDH in the context of the diagnoses and rehabilitation exposure will be important in future analytic methods for outcome prediction.

### Reintegration Back Home

Successful community reengagement includes social, leisure, instrumental, vocational, school or volunteer participation. For some diagnoses like stroke, reengagement in community activities and self-care is best predicted by a supportive living situation (49, 50). In patients with TBI, community reintegration is complex, and strength of associations between SDH and outcomes vary widely. Scoping reviews found that white race, higher education, employment, level of disability and mood/affect contribute to reintegration (51). Conversely, poor housing is a risk factor for moderate-to-severe disability after hospital discharge for stroke (52). SDH are critical in the success of personal and societal engagement over the long-term.

### Outpatient Setting

Common musculoskeletal conditions, such as arthritis and chronic back pain, disproportionately affect people who are non-white (black, Hispanic), older, have less than a high school level of education, low annual income, single, unemployed, and/or living in inner cities or rural areas (53–55). Job positions requiring more craft skills than managerial-professional skills are strongly related to back pain (56). Prospective evidence shows that pain symptom severity and disability are worse over time among non-white, less-educated individuals (26, 56, 57) and those with less social support (24). Neighborhood location and resources may influence effectiveness of long-term care for people in different geographical areas. For example, people with knee osteoarthritis who live in safe areas with better social cohesion and have resources for participation in physical activity have better mental health (25), which may improve health outcomes overall. In a mixed sample of individuals with stroke, cardiopulmonary disease and arthritis, social identification (social group membership in the community) fostered feelings of self-efficacy and confidence, which reduced disability (27). Our understanding of SDH effects on functional outcomes across all settings could be improved with the study of additional determinants related to rehabilitation access, quality and effectiveness. Additional determinants required to fully understand functional outcome trajectories are in Supplementary Table 1.

## Leveraging Big Data and Expanding Machine Learning in Physiatry

An exciting opportunity to improve prediction of functional improvement exists through the use of artificial intelligence. Based on existing evidence and state of the science, various machine learning algorithms already helped create predictive equations for standard functional measures after inpatient rehabilitation for stroke: Functional Independence Measure (FIM), 10-m walk test, 6-min walk test and Berg Balance Scale (58). Moreover, machine-learning modeling predicted 30-day hospital readmissions after discharge to post-acute care, using patient SDH and other characteristics (59).

### Existing Datasets

Current datasets used in physiatry contain a mixture of institutional data obtained by EHR extraction. Specific registries and administrative datasets each have advantages and disadvantages, described Supplementary Table 2. Often, breadth, detail and consistency of data are sacrificed. Outcomes in PM&R are focused on functional outcomes rather than survival, and tracking and recording these data remains a major challenge to expanding datasets.

Many physiatry-specific datasets are focused on specific conditions, such as stroke or osteoarthritis, and contain limited SDH data (Supplementary Table 3). One of the more generalized datasets is the Uniform Data System for Medical Rehabilitation which has existed for almost 30 years and is used by approximately 70% of inpatient rehabilitation facilities in the U.S. and contains FIM data before, during and after completed rehabilitation (60). Similarly, the Model Systems for Burn, TBI and SCI have been in use over 20 years, and gather social, psychologic, functional data and patient outcomes (61). More recently, datasets are being developed which examine patient-reported outcomes for benchmarking Medicare payments. These include the American Academy of Physical Medicine and Rehabilitation registries (for low back pain, ischemic stroke), and the American Spine Registry created by the American Association of Neurologic Surgeons and American Academy of Orthopedic Surgeons (62, 63). SDH tend to be limited to age, gender, race/ethnicity, insurance type, housing situation and discharge location. This highlights the need to expand data collection to create better predictive models. Non-specific datasets (Supplementary Table 3) typically contain the “easy-to-collect” SDH like age, gender, race/ethnicity, insurance type, living situation (housing type, people in household), discharge location and readmissions. Functional status is often assessed by proxy for where the patient was discharged, and readmission to a hospital or another rehabilitation facility (64). Unfortunately, the physical/occupational therapy or rehabilitation type received, and functional performance are generally not present, as seen in the Supplementary Tables 2, 3.

### Extraction of SDH, Rehabilitation Components and Key Words

Often, research does not present the rehabilitation elements or different proportions of time spent in specific activities like gait retraining, patient education or activities of daily living (65). The use of large datasets with detailed information about therapeutic activities and outcomes including SDH, functional assessment scores, and patient-reported outcome measures could improve treatment precision and optimize patient success. Natural language processing (NLP), language modeling and word embedding techniques could be used on provider notes to find items from patient interactions or audio files that are related to SDH and functionality (66). For example, NLP can be used to identify which patients are more likely to miss therapy, or functional recovery time could be predicted for resource allocation and treatment planning (67), as well as identify SDH impact on functional progress among physiatric patients.

### Non-linear Modeling of Functional Change

**Figure 2:**
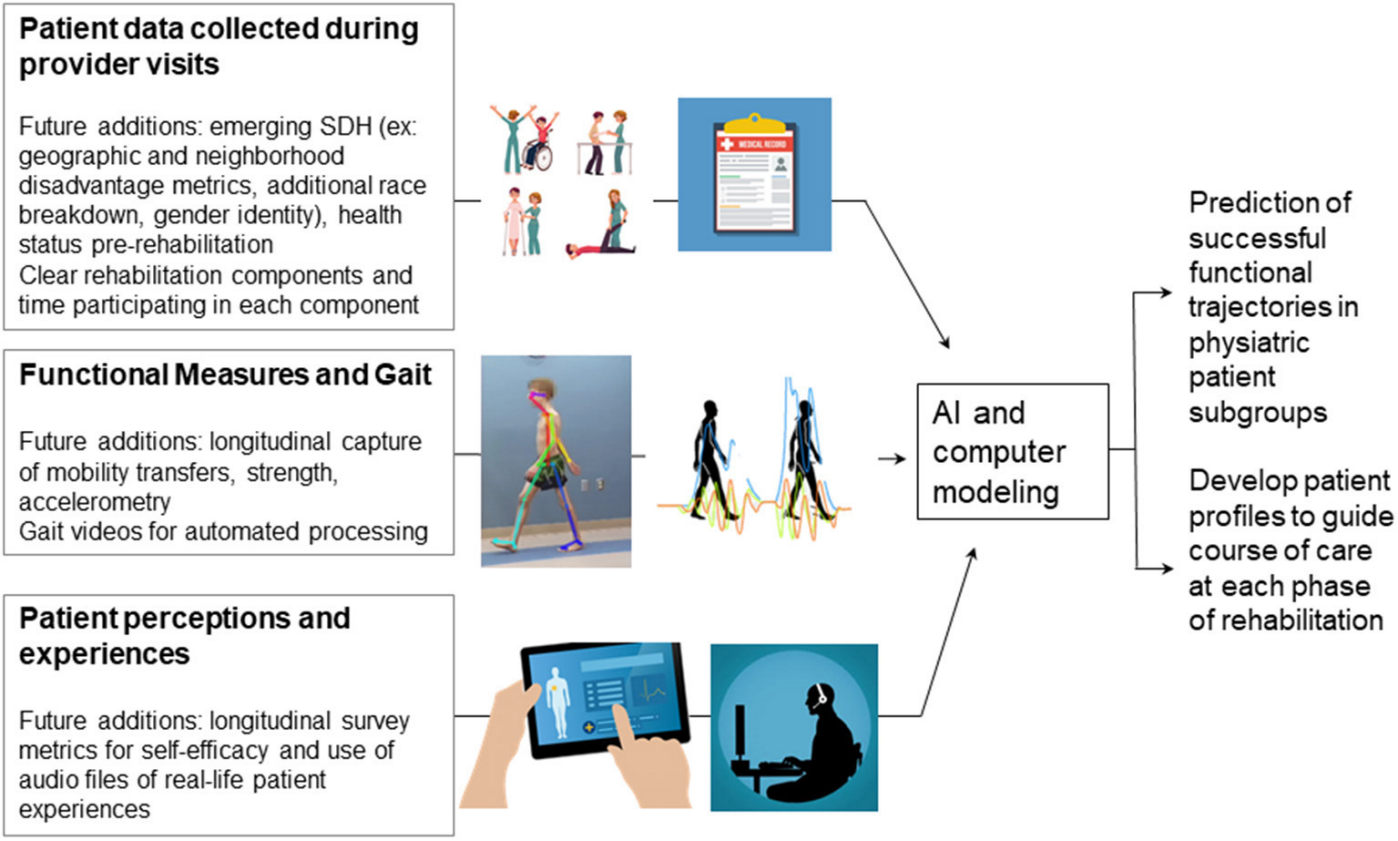
Proposed functional and performance metrics and processes to improve prediction of functional trajectories in patients with physiatric diagnoses. SDH, Social determinants of health; AI, Artificial intelligence.

Functional recovery in physiatry is rarely a linear process. Patients initiating care at lower functional levels receive more treatment, and more treatment is associated with longer recovery, likely because treatment was resourced according to need (68). This can be addressed by using non-linear modeling using supervised techniques such as non-linear regression, decision trees, non-linear support vector machines and unsupervised techniques like clustering and artificial neural networks (69). For example, non-linear modeling was applied to create a non-linear risk score for stroke which performed better than the Framingham Stroke Risk Score, and we postulate that this approach would also be successful in predicting functional outcomes following stroke or other diseases in the early or later recovery stages (70, 71). Furthermore, the effects of SDH on functional outcomes in physiatry is unlikely to be linear and their inclusion could have protective effects against health plan underpayment for treatment in high-risk vulnerable populations (72). In our view, non-linear modeling methods would help the field better establish which SDH impact which aspects of functionality during each stage of rehabilitation from acute to long-term. These techniques could immediately and positively change how treatment is applied to different patient diagnoses depending on the acuity of the condition. Figure 2 provides a summary of these novel techniques.

## Proposed Novel Measurement Approaches 194

Several challenges exist with interpretation of functional outcomes in physiatry. First, the level of functional impairment dictates the type and amount physiatry services provided and the long-term outcomes independent from SDH. Second, the health status (defined as comorbid health conditions, personal, social and environmental factors) preceding hospital admission or outpatient visit impacts rehabilitation outcomes. Physical function and mobility are embedded in many health measures, from post-acute care and surgical outcomes, to chronic frailty and disability; these are represented as a domain of human activity in the International Classification of Functioning, Disability, and Health (73). Yet, mobility and other functional activities remain under-studied, and commonly-used medical terminologies do not reflect functional status in the EHR. Health status impacts FIM scores, is linked to SDH and can be used for clinical decision-making or predicting functional outcomes. For example, gender-related differences exist in the health status factors that result in worse functional status after TBI for men (dementia, epilepsy, chronic cardiovascular pathology, mental health disorders) (74). Third, changes in SDH over time are rarely accounted for in physiatry research, a critical flaw that has been a barrier to understanding changes in function with different treatment approaches. Thus, linking SDH longitudinally to patient data and function is the next essential step in advancing treatment precision.

### Approach 1. Establishing Functional Level and Health Status Prior to Disease

Physiatrists should have data regarding the patient's general health status and functional level prior to disease onset and be able to use these data to predict the extent of the patient's potential for recovery. These data would ideally be obtained prior to the disease, possibly at prior primary care physician visits or collected data from wearable technology such as FitBit^®^. Less optimal methods would be surveying the patient and/or their family and friends regarding their estimation of patient functional capacity.

### Approach 2. Longitudinal Capture of SDH and Physical Function

The level of function at the start of rehabilitation coupled with health status and SDH, shape the trajectory and time-scale of recovery (58, 68). Supportive evidence includes widening disparities in FIM scores after stroke among white, black and Hispanic patients from rehabilitation to 12 months-post discharge; these different recovery patterns are strongly influenced by age (75). Also, there is population shifting among subgroups of patients undergoing physiatric treatment. Compared to years prior, individuals with non-vascular lower limb amputation today are more cognitively intact, but less physically functional and less able to afford prostheses—all of which can impact functional and clinical outcomes independent of other treatments provided (76). Longitudinal capture of SDH and physical function metrics will dramatically improve interpretation of treatment efficacy, disability fluctuation patterns, hospital readmissions, morbidity risk and mortality over time.

### Approach 3. Capturing Movement and Gait Patterns in the EHR

Daily activity metrics that reflect community ambulation and physical activity patterns could be clinically useful to determine real-life functioning in the home and community (77). These metrics could include distance walked, daily step count and intensity of the steps taken; higher intensities are related to lower risk for major mobility disability (78) and predict independent living (79). Commercially-available triaxial accelerometers that produce raw acceleration output (Actigraph, Axivity, GeneActiv) can be used to determine average acceleration, intensity gradient or acceleration above which most active 30 min are captured. These raw data could be uploaded into the EHR on personal medical portals at specific follow-up intervals from the home or clinic.

Movement patterns produced during execution of functional tests provide insight on neuromotor strategies across a diverse range of patients. Gait metrics could be quickly extracted from 2D trajectories of body poses using single camera videos from the sagittal view (computer models available and freely shared) (80). Clinically-meaningful metrics could include gait speed, cadence, gait deviation index and knee flexion. Collection of gait metrics over time as part of routine care, coupled with SDH and clinical measures, would provide a complete picture of the patient experience and success with treatment. For example, lower gait speed was previously associated with age, literacy, and blue collar occupation (81).

### Approach 4. Perceived Functional Outcomes and Self Efficacy

Inclusion of measures of perception of physical function and self-efficacy would inform how much functional limitation is modified by thoughts and feelings. Higher self-efficacy (82) directly relates to better community reintegration (83), functionality (24, 82), and independence in conditions such as amputation and osteoarthritis. Patient-perceived function and self-efficacy could be measured through traditional methods such as survey. We propose a new approach of capturing patient experiences through audio recording analysis. We envision a patient portal (accessible through phone or computer) in which patients could record changes in symptoms, pain and functional ability at specific time points after initiation of treatment or follow-up using standardized prompt questions from validated surveys or a diagnosis-specific question set. These audio files could be uploaded as part of the EHR. Additional free talking could supplement standardized responses and the language analyzed for key words that represent changes in well-being that may not otherwise be captured in EHR. These could include state of emotional well-being (such as, “feeling depressed,” “sad”), SDH (including “lost my job,” “retired,” “moved to new area,” “taking care of my husband,” “got married”) and physical function (examples could include “my knee pain is worse,” can't drive anymore'). These methods could improve understanding of functional fluctuations over time in different patient subgroups.

## Moving Forward

As we move toward precision medicine, physiatry continues to face unique challenges such as insufficient datasets, difficulty with data access-sharing and lack of SDH and functional outcomes. Physiatry is uniquely positioned to: (1) implement new forms of data collection and integration such as movement and gait patterning, and (2) improve collection of SDH and patient-reported outcomes focusing on function. From a health system-wide perspective, we advocate for a consistent and standardized collection of SDH, health status and functional measures over time for diagnoses commonly treated in physiatry. Sources could include patient EHR, surveys, claims data, smart phone applications and wearable devices. Unique sources of data could include subcategories of race, “area deprivation scores” from the Neighborhood Atlas (84), and census tract data. Using artificial intelligence with the sources proposed here could help establish optimal treatment pathways for different patient subgroups, which in turn could improve preparation at each phase of rehabilitation care and treatment precision.

## Author Contributions

All authors listed have made a substantial, direct and intellectual contribution to the work, and approved it for publication.

## Conflict of Interest

The authors declare that the research was conducted in the absence of any commercial or financial relationships that could be construed as a potential conflict of interest.

## Publisher's Note

All claims expressed in this article are solely those of the authors and do not necessarily represent those of their affiliated organizations, or those of the publisher, the editors and the reviewers. Any product that may be evaluated in this article, or claim that may be made by its manufacturer, is not guaranteed or endorsed by the publisher.

## References

[1] Centers for Disease Control. Social Determinants of Health: Know What Affects Health (2021).

[2] Guo Y Chen Z Xu K George TJ Wu Y Hogan W. International classification of diseases, tenth revision, clinical modification social determinants of health codes are poorly used in electronic health records. Medicine. (2020) 99:e23818. 10.1097/MD.0000000000023818 33350768 PMC7769291

[3] Siebens HC Sharkey P Aronow HU Deutscher D Roberts P Munin MC. Variation in rehabilitation treatment patterns for hip fracture treated with arthroplasty. PMR. (2016) 8:191–207. 10.1016/j.pmrj.2015.07.005 26226210

[4] Simões JL Soares S Sa-Couto P Lopes C Magina D Melo E. The influence of presurgical factors on the rehabilitation outcome of patients following hip arthroplasty. Rehabil Nurs. (2019) 44:189–202. 10.1097/rnj.0000000000000126 29369113

[5] Pournajaf S Goffredo M Agosti M Massucci M Ferro S Franceschini M. Community ambulation of stroke survivors at 6 months follow-up: an observational study on sociodemographic and sub-acute clinical indicators. Eur J Phys Rehabil Med. (2019) 55:433–41. 10.23736/S1973-9087.18.05489-8 30543267

[6] Sandel ME Wang H Terdiman J Hoffman JM Ciol MA Sidney S. Disparities in stroke rehabilitation: results of a study in an integrated health system in northern California. PMR. (2009) 1:29–40. 10.1016/j.pmrj.2008.10.012 19627870 PMC3432287

[7] Tooth L McKenna K Goh K Varghese P. Length of stay, discharge destination, and functional improvement: utility of the Australian national subacute and nonacute patient casemix classification. Stroke. (2005) 36:1519–25. 10.1161/01.STR.0000169923.57038.a8 15920028

[8] Meijer R van Limbeek J Kriek B Ihnenfeldt D Vermeulen M de Haan R. Prognostic social factors in the subacute phase after a stroke for the discharge destination from the hospital stroke-unit. A systematic review of the literature. Disabil Rehabil. (2004) 26:191–7. 10.1080/09638280310001636437 15164952

[9] Dhand A Lang CE Luke DA Kim A Li K McCafferty L. Social network mapping and functional recovery within 6 months of ischemic stroke. Neurorehabil Neural Repair. (2019) 33:922–32. 10.1177/1545968319872994 31524080 PMC6851478

[10] Hay CC Graham JE Pappadis MR Sander AM Hong I Reistetter TA. The impact of one's sex and social living situation on rehabilitation outcomes after a stroke. Am J Phys Med Rehabil. (2020) 99:48–55. 10.1097/PHM.0000000000001276 31343498 PMC6920562

[11] Kobylańska M Kowalska J Neustein J Mazurek J Wójcik B Bełza M. The role of biopsychosocial factors in the rehabilitation process of individuals with a stroke. Work. (2018) 61:523–35. 10.3233/WOR-162823 30475778 PMC6398539

[12] Ouyang F Wang Y Huang W Chen Y Zhao Y Dang G. Association between socioeconomic status and post-stroke functional outcome in deprived rural southern China: a population-based study. BMC Neurol. (2018) 18:12. 10.1186/s12883-018-1017-4 29370778 PMC5785852

[13] Delbari A Keyghobadi F Momtaz YA Keyghobadi F Akbari R Kamranian H. Sex differences in stroke: a socioeconomic perspective. Clin Interv Aging. (2016) 11:1207–12. 10.2147/CIA.S113302 27660426 PMC5019160

[14] Everink IHJ van Haastregt JCM van Hoof SJM Schols JMGA Kempen GIJM. Factors influencing home discharge after inpatient rehabilitation of older patients: a systematic review. BMC Geriatr. (2016) 16:5. 10.1186/s12877-016-0187-4 26755206 PMC4709872

[15] Hsieh CH DeJong G Groah S Ballard PH Horn SD Tian W. Comparing rehabilitation services and outcomes between older and younger people with spinal cord injury. Arch Phys Med Rehabil. (2013) 94(Suppl. 4):S175–86. 10.1016/j.apmr.2012.10.038 23527773

[16] DeJong G Tian W Hsieh CH Junn C Karam C Ballard PH. Rehospitalization in the first year of traumatic spinal cord injury after discharge from medical rehabilitation. Arch Phys Med Rehabil. (2013) 94(Suppl. 4):S87–97. 10.1016/j.apmr.2012.10.037 23527776

[17] Oyesanya TO Moran TP Espinoza TR Wright DW. Regional variations in rehabilitation outcomes of adult patients with traumatic brain injury: a uniform data system for medical rehabilitation investigation. Arch Phys Med Rehabil. (2021) 102:68–75. 10.1016/j.apmr.2020.07.011 32861669 PMC9284411

[18] Dahdah MN Barnes S Buros A Dubiel R Dunklin C Callender L. Variations in inpatient rehabilitation functional outcomes across centers in the traumatic brain injury model systems study and the influence of demographics and injury severity on patient outcomes. Arch Phys Med Rehabil. (2016) 97:1821–31. 10.1016/j.apmr.2016.05.005 27246623

[19] Graham JE Radice-Neumann DM Reistetter TA Hammond FM Dijkers M Granger CV. Influence of sex and age on inpatient rehabilitation outcomes among older adults with traumatic brain injury. Arch Phys Med Rehabil. (2010) 91:43–50. 10.1016/j.apmr.2009.09.017 20103395 PMC4148208

[20] Arango-Lasprilla JC Rosenthal M Deluca J Cifu DX Hanks R Komaroff E. Functional outcomes from inpatient rehabilitation after traumatic brain injury: how do Hispanics fare? Arch Phys Med Rehabil. (2007) 88:11–8. 10.1016/j.apmr.2006.10.029 17207669

[21] Arango-Lasprilla JC Ketchum JM Cifu D Hammond F Castillo C Nicholls E. Predictors of extended rehabilitation length of stay after traumatic brain injury. Arch Phys Med Rehabil. (2010) 91:1495–504. 10.1016/j.apmr.2010.07.010 20875505

[22] Venkataraman K Fong NP Chan KM Tan BY Menon E Ee CH. Rehabilitation outcomes after inpatient rehabilitation for lower extremity amputations in patients with diabetes. Arch Phys Med Rehabil. (2016) 97:1473–80. 10.1016/j.apmr.2016.04.009 27178094

[23] Dillingham TR Yacub JN Pezzin LE. Determinants of postacute care discharge destination after dysvascular lower limb amputation. PMR. (2011) 3:336–44. 10.1016/j.pmrj.2010.12.019 21497320 PMC5391794

[24] Miller MJ Cook PF Magnusson DM Morris MA Blatchford PJ Schenkman ML. Self-efficacy and social support are associated with disability for ambulatory prosthesis users after lower-limb amputation. PMR. (2021) 13:453–60. 10.1002/pmrj.12464 32926546 PMC7873129

[25] Kowitt SD Aiello AE Callahan LF Fisher EB Gottfredson NC Jordan JM. How are neighborhood characteristics associated with mental and physical functioning among older adults with radiographic knee osteoarthritis? Arthritis Care Res (Hoboken). (2021) 73:308–17. 10.1002/acr.24125 31841258 PMC7295685

[26] Fliesser M De Witt Huberts J Wippert PM. The choice that matters: the relative influence of socioeconomic status indicators on chronic back pain- a longitudinal study. BMC Health Serv Res. (2017) 17:800. 10.1186/s12913-017-2735-9 29197372 PMC5712136

[27] Cameron JE Voth J Jaglal SB Guilcher SJT Hawker G Salbach NM. “In this together”: Social identification predicts health outcomes (via self-efficacy) in a chronic disease self-management program. Soc Sci Med. (2018) 208:172–9. 10.1016/j.socscimed.2018.03.007 29598988

[28] Pincus T Castrejon I. Low socioeconomic status and patient questionnaires in osteoarthritis: challenges to a “biomedical model” and value of a complementary “biopsychosocial model” Clin Exp Rheumatol. (2019) 120:18–23. 31621564

[29] Odonkor CA Esparza R Flores LE Verduzco-Gutierrez M Escalon MX Solinsky R. Disparities in health care for black patients in physical medicine and rehabilitation in the United States: a narrative review. PMR. (2021) 13:180–203. 10.1002/pmrj.12509 33090686

[30] Longley V Peters S Swarbrick C Bowen A. What factors affect clinical decision-making about access to stroke rehabilitation? A systematic review. Clin Rehabil. (2019) 33:304–16. 10.1177/0269215518808000 30370792 PMC6348456

[31] Labberton AS Barra M Rønning OM Thommessen B Churilov L Cadilhac DA. Patient and service factors associated with referral and admission to inpatient rehabilitation after the acute phase of stroke in Australia and Norway. BMC Health Serv Res. (2019) 19:871. 10.1186/s12913-019-4713-x 31752874 PMC6873491

[32] Ottenbacher KJ Smith PM Illig SB Linn RT Gonzales VA Ostir GV. Disparity in health services and outcomes for persons with hip fracture and lower extremity joint replacement. Med Care. (2003) 41:232–41. 10.1097/01.MLR.0000044902.01597.54 12555051

[33] Benz T Angst F Oesch P Hilfiker R Lehmann S Mueller Mebes C. Comparison of patients in three different rehabilitation settings after knee or hip arthroplasty: a natural observational, prospective study. BMC Musculoskelet Disord. (2015) 16:317. 10.1186/s12891-015-0780-2 26497597 PMC4619418

[34] Freburger JK Holmes GM Ku LJE. Postacute rehabilitation care for hip fracture: who gets the most care? J Am Geriatr Soc. (2012) 60:1929–35. 10.1111/j.1532-5415.2012.04149.x 23036079 PMC3470736

[35] Singh JA Kallan MJ Chen Y Parks ML Ibrahim SA. Association of race/ethnicity with hospital discharge disposition after elective total knee arthroplasty. JAMA Netw Open. (2019) 2:e1914259. 10.1001/jamanetworkopen.2019.14259 31664446 PMC6824220

[36] Vincent HK Vincent KR. Functional and economic outcomes of cardiopulmonary patients: a preliminary comparison of the inpatient rehabilitation and skilled nursing facility environments. Am J Phys Med Rehabil. (2008) 87:371–80. 10.1097/PHM.0b013e31816dd251 18427219

[37] Hong I Goodwin JS Reistetter TA Kuo YF Mallinson T Karmarkar A. Comparison of functional status improvements among patients with stroke receiving postacute care in inpatient rehabilitation vs skilled nursing facilities. JAMA Netw Open. (2019) 2:e1916646. 10.1001/jamanetworkopen.2019.16646 31800069 PMC6902754

[38] Frankel RM Stein T. Getting the most out of the clinical encounter: the four habits model. J Med Pract Manage. (2001) 16:184–91. 11317576

[39] Cifu DX Kreutzer JS Marwitz JH Rosenthal M Englander J High W. Functional outcomes of older adults with traumatic brain injury: a prospective, multicenter analysis. Arch Phys Med Rehabil. (1996) 77:883–8. 10.1016/S0003-9993(96)90274-9 8822678

[40] Jourdan C Bayen E Darnoux E Ghout I Azerad S Ruet A. Patterns of post-acute health care utilization after a severe traumatic brain injury: Results from the PariS-TBI cohort. Brain Inj. (2015) 29:701–8. 10.3109/02699052.2015.1004646 25789712

[41] Garcia JJ Warren KL. Race/ethnicity matters: differences in poststroke inpatient rehabilitation outcomes. Ethn Dis. (2019) 29:599–608. 10.18865/ed.29.4.599 31641327 PMC6802163

[42] Zhang X Qiu H Liu S Li J Zhou M. Prediction of prolonged length of stay for stroke patients on admission for inpatient rehabilitation based on the International Classification of Functioning, Disability, and Health (ICF) generic set: a study from 50 centers in China. Med Sci Monit. (2020) 26:e918811. 10.12659/MSM.918811 31901931 PMC6977619

[43] Cations M Lang C Crotty M Wesselingh S Whitehead C Inacio MC. Factors associated with success in transition care services among older people in Australia. BMC Geriatr. (2020) 20:496. 10.1186/s12877-020-01914-z 33228558 PMC7686713

[44] Scrutinio D Battista P Guida P Lanzillo B Tortelli R. Sex differences in long-term mortality and functional outcome after rehabilitation in patients with severe stroke. Front Neurol. (2020) 11:84. 10.3389/fneur.2020.00084 32132967 PMC7040356

[45] Vincent HK Alfano AP Lee L Vincent KR. Sex and age effects on outcomes of total hip arthroplasty after inpatient rehabilitation. Arch Phys Med Rehabil. (2006) 87:461–7. 10.1016/j.apmr.2006.01.002 16571383

[46] Flanagan NM Rizzo VM James GD Spegman A Barnawi NA. Predicting risk factors for 30-day readmissions following discharge from post-acute care. Prof Case Manag. (2018) 23:139–46. 10.1097/NCM.0000000000000261 29601425

[47] Canori A Kumar A Hiremath SV. Factors associated with multiple hospital readmissions for individuals with spinal cord injury. Commonhealth (Phila). (2020) 1:57–61. 10.15367/ch.v1i2.399 33554212 PMC7861491

[48] Cardenas DD Hoffman JM Kirshblum S McKinley W. Etiology and incidence of rehospitalization after traumatic spinal cord injury: a multicenter analysis. Arch Phys Med Rehabil. (2004) 85:1757–63. 10.1016/j.apmr.2004.03.016 15520970

[49] Erler KS Sullivan V Mckinnon S Inzana R. Social support as a predictor of community participation after stroke. Front Neurol. (2019) 10:1013. 10.3389/fneur.2019.01013 31616364 PMC6763952

[50] Elloker T Rhoda AJ. The relationship between social support and participation in stroke: a systematic review. Afr J Disabil. (2018) 7:357. 10.4102/ajod.v7i0.357 30349808 PMC6191741

[51] Kersey J Terhorst L Wu CY Skidmore E. A scoping review of predictors of community integration following traumatic brain injury: a search for meaningful associations. J Head Trauma Rehabil. (2019) 34:E32–41. 10.1097/HTR.0000000000000442 30499925

[52] de Villiers L Badri M Ferreira M Bryer A. Stroke outcomes in a socio-economically disadvantaged urban community. S Afr Med J. (2011) 101:345–8. 10.7196/SAMJ.4588 21837880

[53] Guillemin F Carruthers E Li LC. Determinants of MSK health and disability–social determinants of inequities in MSK health. Best Pract Res Clin Rheumatol. (2014) 28:411–33. 10.1016/j.berh.2014.08.001 25481424

[54] Vennu V Abdulrahman TA Alenazi AM Bindawas SM. Associations between social determinants and the presence of chronic diseases: data from the osteoarthritis Initiative. BMC Public Health. (2020) 20:1323. 10.1186/s12889-020-09451-5 32867751 PMC7461338

[55] Jonsdottir S Ahmed H Tómasson K Carter B. Factors associated with chronic and acute back pain in wales, a cross-sectional study. BMC Musculoskelet Disord. (2019) 20:215. 10.1186/s12891-019-2477-4 31092222 PMC6521348

[56] Fliesser M De Witt Huberts J Wippert PM. Education, job position, income or multidimensional indices? Associations between different socioeconomic status indicators and chronic low back pain in a German sample: a longitudinal field study. BMJ Open. (2018) 8:e020207. 10.1136/bmjopen-2017-020207 29705759 PMC5931294

[57] Allen KD Helmick CG Schwartz TA DeVellis RF Renner JB Jordan JM. Racial differences in self-reported pain and function among individuals with radiographic hip and knee osteoarthritis: the Johnston County Osteoarthritis Project. Osteoarthritis Cartilage. (2009) 17:1132–6. 10.1016/j.joca.2009.03.003 19327733 PMC2731003

[58] Harari Y O'Brien MK Lieber RL Jayaraman A. Inpatient stroke rehabilitation: prediction of clinical outcomes using a machine-learning approach. J Neuroeng Rehabil. (2020) 17:71. 10.1186/s12984-020-00704-3 32522242 PMC7288489

[59] Howard EP Morris JN Schachter E Schwarzkopf R Shepard N Buchanan ER. Machine-learning modeling to predict hospital readmission following discharge to post-acute care. J Am Med Dir Assoc. (2021) 22:1067–72.e29. 10.1016/j.jamda.2020.12.017 33454309

[60] Graham JE Granger CV Karmarkar AM Deutsch A Niewczyk P Divita MA. The uniform data system for medical rehabilitation: report of follow-up information on patients discharged from inpatient rehabilitation programs in 2002-2010. Am J Phys Med Rehabil. (2014) 93:231–44. 10.1097/PHM.0b013e3182a92c58 24088780 PMC3944381

[61] Model Systems Knowledge Translation Center. Directry of Model Systems. (2021). Available online at: https://msktc.org/sci/model-system-centers (accessed July 7, 2021).

[62] American Academy of Physical Medicine and Rehabilitation. Patient Reported Outcomes Module. (2021). Available online at: https://www.aapmr.org/quality-practice/registry/patient-reported-outcomes (accessed July 5, 2021).

[63] American Spine Registry. The National Quality Improvement Registry for Spine Care. (2021). Available online at: https://www.americanspineregistry.org/ (accessed July 1, 2021).

[64] Meddings J Reichert H Smith SN Iwashyna TJ Langa KM Hofer TP. The impact of disability and social determinants of health on condition-specific readmissions beyond Medicare risk adjustments: a cohort study. J Gen Intern Med. (2017) 32:71–80. 10.1007/s11606-016-3869-x 27848189 PMC5215164

[65] Schumacher R Müri RM Walder B. Integrated health care management of moderate to severe TBI in older patients-a narrative review. Curr Neurol Neurosci Rep. (2017) 17:92. 10.1007/s11910-017-0801-7 28986740

[66] Reeves RM Christensen L Brown JR Conway M Levis M Gobbel GT. Adaptation of an NLP system to a new healthcare environment to identify social determinants of health. J Biomed Inform. (2021) 120:103851. 10.1016/j.jbi.2021.103851 34174396 PMC8386129

[67] Ong CJ Orfanoudaki A Zhang R Caprasse FPM Hutch M Ma L. Machine learning and natural language processing methods to identify ischemic stroke, acuity and location from radiology reports. PLoS ONE. (2020) 15:e0234908. 10.1371/journal.pone.0234908 32559211 PMC7304623

[68] Hart T Kozlowski AJ Whyte J Poulsen I Kristensen K Nordenbo A. Functional recovery after severe traumatic brain injury: an individual growth curve approach. Arch Phys Med Rehabil. (2014) 95:2103–10. 10.1016/j.apmr.2014.07.001 25010537

[69] Chatterjee P Cymberknop LJ Armentano R. Nonlinear systems in healthcare towards intelligent disease prediction. In: Nonlinear Systems- Theoretical Aspects and Recent Applications. Intech Open (2019). 10.5772/intechopen.88163

[70] Orfanoudaki A Chesley E Cadisch C Stein B Nouh A Alberts MJ. Machine learning provides evidence that stroke risk is not linear: the non-linear Framingham stroke risk score. PLoS ONE. (2020) 15:e0232414. 10.1371/journal.pone.0232414 32437368 PMC7241753

[71] Kwakkel G Kollen BJ. Predicting activities after stroke: what is clinically relevant? Int J Stroke. (2013) 8:25–32. 10.1111/j.1747-4949.2012.00967.x 23280266

[72] Irvin JA Kondrich AA Ko M Rajpurkar P Haghgoo B Landon BE. Incorporating machine learning and social determinants of health indicators into prospective risk adjustment for health plan payments. BMC Public Health. (2020) 20:608. 10.1186/s12889-020-08735-0 32357871 PMC7195714

[73] Newman-Griffis D Fosler-Lussier E. Automated coding of under-studied medical concept domains: linking physical activity reports to the International Classification of Functioning, Disability, and Health. Front Digit Health. (2021) 3:620828. 10.3389/fdgth.2021.620828 33791684 PMC8009547

[74] Chan V Sutton M Mollayeva T Escobar MD Hurst M Colantonio A. Data mining to understand how health status preceding traumatic brain injury affects functional outcome: a population-based sex-stratified study. Arch Phys Med Rehabil. (2020) 101:1523–31. 10.1016/j.apmr.2020.05.017 32544398 PMC7483900

[75] Simmonds KP Luo Z Reeves M. Race/ethnic and stroke subtype differences in poststroke functional recovery after acute rehabilitation. Arch Phys Med Rehabil. (2021) 102:1473–81. 10.1016/j.apmr.2021.01.090 33684363

[76] Batten H Kuys S McPhail S Varghese P Mandrusiak A. Are people with lower limb amputation changing? A seven-year analysis of patient characteristics at admission to inpatient rehabilitation and at discharge. Disabil Rehabil. (2019) 41:3203–9. 10.1080/09638288.2018.1492033 30182758

[77] Gothe NP Bourbeau K. Associations between physical activity intensities and physical function in stroke survivors. Am J Phys Med Rehabil. (2020) 99:733–8. 10.1097/PHM.0000000000001410 32167953

[78] Fanning J Rejeski WJ Chen SH Nicklas BJ Walkup MP Axtell RS. A case for promoting movement medicine: preventing disability in the LIFE Randomized Controlled Trial. J Gerontol A Biol Sci Med Sci. (2019) 74:1821–7. 10.1093/gerona/glz050 30778518 PMC6777081

[79] Dunlop DD Song J Hootman JM Nevitt MC Semanik PA Lee J. One hour a week: moving to prevent disability in adults with lower extremity joint symptoms. Am J Prev Med. (2019) 56:664–72. 10.1016/j.amepre.2018.12.017 30902564 PMC6475497

[80] Kidziński Ł Yang B Hicks JL Rajagopal A Delp SL Schwartz MH. Deep neural networks enable quantitative movement analysis using single-camera videos. Nat Commun. (2020) 11:4054. 10.1038/s41467-020-17807-z 32792511 PMC7426855

[81] Busch T de A Duarte YA Pires Nunes D Lebrão ML Satya Naslavsky M dos Santos Rodrigues A. Factors associated with lower gait speed among the elderly living in a developing country: a cross-sectional population-based study. BMC Geriatr. (2015) 15:35. 10.1186/s12877-015-0031-2 25880124 PMC4391309

[82] Jackson T Xu T Jia X. Arthritis self-efficacy beliefs and functioning among osteoarthritis and rheumatoid arthritis patients: a meta-analytic review. Rheumatology. (2020) 59:948–58. 10.1093/rheumatology/kez219 31211379

[83] Gupta S Jaiswal A Norman K DePaul V. Heterogeneity and its impact on rehabilitation outcomes and interventions for community reintegration in people with spinal cord injuries: an integrative review. Top Spinal Cord Inj Rehabil. (2019) 25:164–85. 10.1310/sci2502-164 31068748 PMC6496968

[84] Kind AJH Buckingham WR. Making neighborhood-disadvantage metrics accessible - the neighborhood Atlas. N Engl J Med. (2018) 378:2456–8. 10.1056/NEJMp1802313 29949490 PMC6051533

