# Supplementary material for: Social Determinants of Health in Physiatry: Challenges and Opportunities for Clinical Decision Making and Improving Treatment Precision
Source: Front Public Health. 2021 Nov 11;9:738253. doi: 10.3389/fpubh.2021.738253 (PMC8632538; doi:10.3389/fpubh.2021.738253)
Supplement: Supplementary file 1 [file Table_1.DOCX]

Supplemental Table 1. Social determinants of health (SDH) commonly assessed in the different phases of physiatric care.

**Setting Common SDH**

Consult Service in an Acute Hospital Gender, age, race/ethnicity

Insurance

Marital status, social or family support

Employment, disability status

Geographic location, rural or urban

Postacute Care Gender, age, race/ethnicity

Insurance

Employment

Provider of child care or other family member

Marital status, social or family support

Housing status (living at home, type of home environment)

Education

Continuity Clinics and Community Gender, age, race/ethnicity

Insurance

**Additional SDH necessary to fully understand functional outcome trajectories in physiatry**

Neighborhood disadvantage metrics Reflects: access to food, safety, education, health

behaviors and stress levels

Granularity of race Using more specific racial categories. (Example: Asian race includes people of Korean, Japanese, Chinese, Vietnamese, and Indonesian descent)

Gender identity Adding categories beyond the male/female gender

identity (transgender, gender neutral, other)

Working life conditions Reflects: safety and hazards, job security, type of health risk exposures, type of duties, ergonomics, bonded or child labor

Early childhood development Represented by: early life stress, relationships with

parents and caregivers, access to early education programs or booster programs

Social inclusion and non-discrimination Represented by: opportunities for human

development, cohesiveness of living conditions, adequacy of social services, quality of civic engagement
