# Supplementary material for: Social Determinants of Health in Physiatry: Challenges and Opportunities for Clinical Decision Making and Improving Treatment Precision
Source: Front Public Health. 2021 Nov 11;9:738253. doi: 10.3389/fpubh.2021.738253 (PMC8632538; doi:10.3389/fpubh.2021.738253)
Supplement: Supplementary file 2 [file Table_2.DOCX]

Supplemental Table 2. Advantages and disadvantages of datasets for understanding social determinants of health (SDH) and predicting functional outcomes in physiatry.

Advantages Disadvantages

Electronic Health Readily-available Specificity required for research

Record Single or multi-institutional approvals to obtain data

SDH may be available May have multiple electronic

Multiple provider insight about health records across settings or patient in record within health system

Multiple providers collecting and entering data

Rarely includes measures of function in standardized way

SDH may requires extensive time to collect from various sources in record

Free hand text difficult to analyze

Registries Single or multi-institutional Requires advance planning to choose

Easy to compare results across variables of interest

analyses and institutions Data may be limited or incomplete

Data are curated Addition of new variables may be

Standardized data collection difficult retrospectively

procedures Data ownership challenges

Functional outcomes may be limited or easy-to-perform

May require moderate to high cost to

participate as a site or to access data

Administrative Easy to study rare occurrences Data may be inaccurate or

Data or cases incomplete

Large volumes of data may be Selection bias

available High cost

Functional measures or SDH may

be very limited
