# Supplementary material for: Social Determinants of Health in Physiatry: Challenges and Opportunities for Clinical Decision Making and Improving Treatment Precision
Source: Front Public Health. 2021 Nov 11;9:738253. doi: 10.3389/fpubh.2021.738253 (PMC8632538; doi:10.3389/fpubh.2021.738253)
Supplement: Supplementary file 3 [file Table_3.DOCX]

Supplemental Table 3. Examples of physiatry-specific and non-specific datasets that include social determinants of heath (SDH) and functional measures.

Sample Databases for SPECIFIC PM&R related diagnoses

- American Academy of Physical Medicine and Rehabilitation (AAPMR); longitudinal design

Includes diagnoses of ischemic stroke, lower back pain; Data are collected from participating centers and includes patient reported outcomes

SDH available: obtained from the medical record

Access: Requires annual fee to participate with data sharing and use

- American Spine Registry; longitudinal design

Incudes cervical and lumbar degenerative spine conditions resulting in surgery; Includes patient reported outcomes, Medicare data is integrated

SDH available: Gender, age, race/ethnicity

Access: Participating sites

- Model Systems National Institute of Disability, Independent Living and Rehabilitation Research (NIDILRR); longitudinal design

Includes diagnoses of Traumatic Brain Injury, Spinal Cord Injury and Burn; includes the FIM instrument, CARE Functional Abilities, quality of life data, disability rating scales

SDH available: Gender, age, race/ethnicity, marital status, insurance, job type, re-hospitalizations, living location, support in home

Access: De-identified data are public, limited use datasets are available per request

- Cerebral Palsy Registry; longitudinal design; diagnosis specific to cerebral palsy. Data include Gross Motor Classification Functional Scale assessment, any related interventions including physical and occupational therapies

SDH available: Gender, age, race, ethnicity, living situation

Access: Data available after IRB review and review by the cerebral palsy research committee

- Osteoarthritis Initiative (OAI); longitudinal study design. Four data collection institutions participated. Data include objective and subjective functional measures, quality of life data, pain, disability ratings, other joint pain sites, imaging, activity counts, labwork

SDH available: Gender, age, race, ethnicity, living situation family history, income, education, insurance, work status

Access: De-identified data are public and datasets are available per request and registration

Sample Databases for ANY PM&R related diagnoses

- The Uniform Data System for Medical Rehabilitation (UDSMR); longitudinal design

An estimated 70% of inpatient rehabilitation facilities in the U.S. contribute data, Functional Independence Measure (FIM) instrument; and includes data from adult and pediatric populations receiving care from any condition leading to rehabilitation

SDH available: Gender, age, race/ethnicity, marital status, insurance type, support in living environment, discharge location, living setting, work status

Access: subscribing facilities or data use agreements

- Healthcare Cost and Utilization Project (HCUP); cross-sectional design (design varies by sub database)

Includes Nationwide Inpatient Sample (NIS), Kids' Inpatient Database (KID), Nationwide Ambulatory Surgery Sample (NASS), Nationwide Emergency Department Sample (NEDS), Nationwide Readmissions Database (NRD)

SDH available: Gender, age, race/ethnicity, insurance, discharge location, median income for the patient’s zip code

Access: Free limited access query system, with more extensive data available for purchase

- National Ambulatory Medical Care Survey (NAMCS) and National Hospital Ambulatory Medical Care Survey (NHAMCS): cross-sectional design, Annual survey: 500 nationally representative hospitals over a 4-week period; Treatment information available. Some variables may require a research proposal

SDH available: Gender, age, race, ethnicity insurance, discharge location

Access: Free to access through the CDC website

- National Health and Nutrition Examination Survey (NHANES): cross-sectional design; Data sources include personal interviews, physical examinations, laboratory tests, nutritional assessment, DNA repository; Among wide battery of objective and subjective measures, includes balance, aerobic fitness, leg and handgrip strength, physical activity monitoring, subjective health rating and physical activity patterns and physical function

SDH available: Gender, age, race, ethnicity, family income, household sizes, education, marital status, military status, occupation, country of birth, citizenship, years in U.S., food security, access to care

Access: Free to access through the CDC website
